# The human microbiome in evolution

**DOI:** 10.1186/s12915-017-0454-7

**Published:** 2017-12-27

**Authors:** Emily R. Davenport, Jon G. Sanders, Se Jin Song, Katherine R. Amato, Andrew G. Clark, Rob Knight

**Affiliations:** 1 Department of Molecular Biology and Genetics, Cornell University, Ithaca, NY USA; 2 Department of Pediatrics, University of California San Diego, La Jolla, CA USA; 3 Department of Anthropology, Northwestern University, Evanston, IL USA; 4 Department of Computer Science & Engineering, University of California San Diego, La Jolla, CA USA; 5 Center for Microbiome Innovation, University of California San Diego, La Jolla, CA USA

**Keywords:** Microbiome, Evolution, Codiversification, Habitat filtering, Transmission

## Abstract

The trillions of microbes living in the gut—the gut microbiota—play an important role in human biology and disease. While much has been done to explore its diversity, a full understanding of our microbiomes demands an evolutionary perspective. In this review, we compare microbiomes from human populations, placing them in the context of microbes from humanity’s near and distant animal relatives. We discuss potential mechanisms to generate host-specific microbiome configurations and the consequences of disrupting those configurations. Finally, we propose that this broader phylogenetic perspective is useful for understanding the mechanisms underlying human–microbiome interactions.

## The microbiome in the context of evolution

We are in the midst of a revolution in our understanding of the human **microbiome** (Box 1). A decade ago, very little was known of the inventory of microbes that inhabit different parts of the human body, how they assemble into communities of varying levels of complexity, and how they relate to microbiomes in other species. Recent improvements in technology for collecting and analyzing DNA sequence data make these questions accessible for the first time. Various aspects of the microbiome have been correlated to a surprising number of human diseases [1], and some microbiome-centric interventions have shown extraordinary efficacy in treatment of specific disorders like recurrent *Clostridium difficile* infection [2]. More broadly, this wealth of additional data has drawn attention to the connections between microbes and foundational elements of organismal biology and ecology [3]. Given the rush to apply knowledge of the human microbiome in fields ranging from medicine to forensics, there has been a heavy emphasis on practical applications of microbiome sequencing, largely from North American and European populations. However, as in many other areas of biology, a true understanding of these patterns and processes requires an evolutionary perspective.

In this review, we focus on the gut microbiome, as surprisingly little comparative data are available for other body sites across human populations or other species. We place the human gut microbiome into its evolutionary context across different modern populations with varying diets, lifestyles, and environmental exposures, and relate evolutionary patterns in the gut microbiome across the mammals to address several key questions. How do human microbiomes compare with our evolutionary ancestors? Are those patterns consistent with a common assumption that the microbiome evolves with the host? If so, what exactly is evolving in a microbiome and how? Does evolutionary history in the microbiome matter to human health and fitness? If so, how can we use evolutionary history to better understand the assembly and effects of the microbiome to the benefit of human health?

To address these questions, we first describe the human gut microbiome and its contents. We then place this information in the context of our closest relatives, the primates, and more distant relatives, mammals. Then we discuss the consequences of modifying species-specific microbiomes, and some of the mechanisms underlying this process. Finally, we point the way to microbiome studies that can better take into account the evolutionary properties of hosts, microbes, and their symbioses to improve our knowledge both of the underlying biology and for practical applications in human and animal health.

## Global diversity of the human microbiome

Although the 6.5 meter human digestive tract consists of three organs—the stomach, small intestine, and large intestine—most human microbiome research focuses on the microbial community (the **microbiota**) of the large intestine as read out through the stool. This community harbors by far the greatest microbial biomass of any organ or surface of the human body. Each milliliter of the large intestine holds approximately 10^11^ microbial cells compared to 10^8^ cells in the small intestine [4]. Typically, researchers turn to non-invasive fecal samples as proxies for the distal colon microbiome. These samples contain a mixture of microbes and human colonocytes from along the length of the digestive tract and have a similar, albeit distinct, composition to intestinal biopsies [5, 6].

**Fig. 1.**
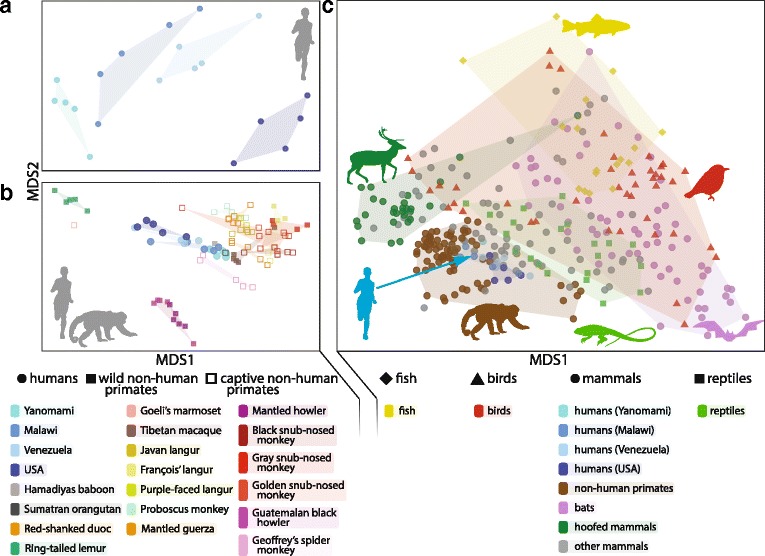
The human gut microbiome within the context of populations and deeper evolutionary landscapes. **a** The microbiomes of different human populations are distinct from each other, especially between industrialized populations such as in the USA and remote, non-industrialized populations such as Malawians or the Guahibo and Yanomami people of the Amazon [14, 17]. **b** Within the context of the greater primates lineage, these differences between human populations become smaller and a connection between humans and captive populations of non-human primates can be seen. **c** Zooming out to include other vertebrate lineages further diminishes those differences, as the effects of deep evolutionary splits between host species and lifestyle characteristics on the gut microbiome become evident. Methods: All data were drawn from publically available studies in Qiita (https://qiita.ucsd.edu/; studies 850, 894, 940, 963, 1056, 1696, 1734, 1736, 1747, 1773, 2182, 2259, 2300, 10052, 10171, 10315, 10376, 10407, 10522). Sequence data for all samples were generated using the same protocol [134] and sequenced on an Illumina MiSeq or HiSeq platform. Sequence data were trimmed to 100 nucleotides and OTUs were picked using the deblur method [135]. Up to five samples per species were randomly selected, rarefied to 10,000 sequences per sample, and unweighted UniFrac [136] distances between samples were computed using Qiime 1.9.1 [137]. The non-metric multidimensional scaling ordination technique was employed in R 3.3.3 [138] to visualize these distances. Silhouettes of the running woman, primate, bird, and bat in **c** are designed by Vexels.com and reproduced with permission

Zooming in to the microbiome of a single individual, an estimated 150 to 400 species reside in each person’s gut based on culture-dependent and -independent techniques [7]. Typically, most of these species belong to the *Bacteroidetes*, *Firmicutes*, *Actinobacteria*, and *Proteobacteria* phyla. The relative proportions of each of these taxa vary dramatically [7] between individuals [8] and even within an individual over the course of their lives [9–11]. Although each person’s microbiome is unique, several trends emerge when we examine microbiomes of populations around the globe (Fig. 1a). Most of what we know of the microbiome comes from studies that examine individuals from highly industrialized and developed (“westernized”) nations, including both medical microbiome research and major microbiome surveys, like the United States focused Human Microbiome Project [8] and the European MetaHIT [12]. However, Western microbiomes differ in several ways from the non-Western microbiomes profiled to date [13–22].

First, Western microbiomes consist of 15 to 30% fewer species than non-Western microbiomes [14, 18, 19]. One proposal, the disappearing microbiome hypothesis, puts forth that technological and cultural changes accompanying industrialization lead to a “disappearing microbiome” [23]. In lieu of building a time machine, the one way to evaluate this hypothesis is to turn to ancient DNA (Box 2).

Second, Western microbiomes lack certain species that consistently occur in non-Western microbiomes. The most striking example is the spiral bacteria in the genus *Treponema*, which appears in the stool of numerous non-Western populations who utilize different subsistence strategies, including hunting and gathering and agriculture [13, 15, 19].

Finally, the relative abundances of common phyla shift between Western and non-Western microbiomes. Western microbiomes generally bear a greater amount of *Bacteroides*, while non-Western microbiomes generally contain greater amounts of Firmicutes and Proteobacteria [15, 17], although exceptions to this trend exist [13]. Taken together, these studies point to the fact that there is no single “human microbiome”, but rather a wide range of configurations that our commensal microbiomes assume.

Given this observation, two key questions arise. First, why do these differences exist between populations? One explanation points to cultural and environmental factors. Diets in particular vary dramatically between cultures and continents. In general, the increased fiber and decreased sugar, fat, and meat in non-Western diets is thought to promote bacterial richness in the gut [13, 24–26]. Additionally, differences in hygiene and medicine likely contribute, including exposure to animals and other septic environments, overuse of antibiotics early in life [23, 27, 28], and differences in enteric parasite carriage in Western populations [20, 29, 30]. Alternatively, portions of the microbiome may simply have diverged along with human populations as they expanded around the globe. For example, the modern day distribution of *Helicobacter pylori* strains aligns with known human migrations [9, 31].

The second question that arises is: Do these differences between populations matter? Are they larger than expected for a species that eats diets and lives in environments as variable as our own? To answer this, it is useful to compare human microbiomes to our close evolutionary relatives, the non-human primates.

## The human gut microbiome in the context of our closest evolutionary ancestors: non-human primates

Despite the evolutionary relevance, surprisingly few studies systematically compare human and non-human primate gut microbiomes. Directly comparing primate and human microbiomes offers insights into what factors shaped our microbiome throughout our evolutionary past. The data that exist demonstrate that, in contrast to our most recent common ancestors, African apes, humans have lower gut microbiota diversity, increased relative abundances of *Bacteroides*, and reduced relative abundances of *Methanobrevibacter* and *Fibrobacter* [32, 33]. Many of these traits are associated with carnivory in other mammals, suggesting that a human dietary shift toward meat-eating over evolutionary timescales may have been accompanied by associated gut microbial shifts [34, 35]. Comparing primate and human microbiomes also provides an indication of how quickly the human microbiome is changing. The human gut microbiome composition appears to have diverged from the ancestral state at an accelerated pace compared to that of the great apes [33]. Some of the hallmarks of human evolution and history potentially responsible include cooked food, the advent of agriculture, population size and density increases, and physiological changes such as the human-specific loss of N-glycolylneuraminic acid (Neu5Gc).

A meta-analysis of non-human primate and human gut microbiome datasets currently available in the Qiita public repository provides some additional insight (see Fig. 1). Human inter-population differences appear similar to the inter-species differences in non-human primates (Fig. 1b). Human inter-population differences are commonly attributed to diet [14, 15]. Similarly, non-human primate gut microbiomes change in response to host habitat and season [36–43], effects which appear to be most strongly linked to spatial and temporal variation in diet. However, differences in gut microbiome composition among non-human primates mirrors host phylogenetic relationships, a pattern known as **phylosymbiosis**, and this signal of host phylogeny persists across a range of timescales, regardless of diet [43]. The human microbiome also exhibits signs of phylosymbiosis. Across primates, human microbiome composition is most similar to Old World monkeys and apes, and distinct from the gut microbiome of New World primates and lemurs (Fig. 1b). Nevertheless, more extensive sampling of non-human primate populations would help determine if the range of variation in human microbiomes is similar to that of non-human primates and if patterns of phylosymbiosis are truly differentially resistant to host environmental context. These data would offer insight into whether unique and/or divergently evolved aspects of human physiology and environments resulted in human-specific gut microbiome traits and whether non-human primates represent a model for understanding dietary transitions and their impact on the microbiome over human evolutionary history.

In this sense, studies of captive primates with artificially manipulated diets provide helpful context for understanding human dietary transitions. Several studies including our own find that captive primates consume less diverse, lower-fiber diets compared to their wild counterparts [42, 44, 45], mirroring the gradual transition to low-fiber diets over the course of human evolution and the stark contrast of modern Western and non-Western diets. One study reports that the low-fiber captive diet provided to howler monkeys and douc langurs results in a “humanization” of the gut microbiome, as characterized by the loss of microbial diversity [41, 44]. However, even with the low-fiber diet, the howler and douc microbiomes were more similar to non-Western than Western human microbiomes, indicating that the relationship between host diet and the gut microbiota differs between human and non-human primates when considering specific microbial taxa. Our own study comparing the gut microbiomes of vervets and humans consuming both high- and low-fiber diets reports similar results [41]. In contrast to observations of Western versus non-Western human populations [14], *Bacteroides* relative abundances are lower in captive animals with low-fiber diets [44], while *Prevotella* relative abundances are higher. These data indicate that closely related microbial taxa may have evolved to encode different metabolic functions in humans and non-human primate microbiomes. Given the overall similarities among primate microbiomes, targeting these related but contrasting lineages for more detailed genomic and functional characterization offers unique opportunities for understanding both the overall function of the human microbiome as well as how evolution of its constituents impacts human health.

## The human gut microbiome in the context of more distant ancestors: mammals

The association between diet and phylogeny among primate microbiomes echoes the larger patterns of diversity among vertebrate gut communities, as evidenced by looking at convergence of dietary transitions and microbiomes across mammals (Fig. 1c) [34, 35]. Transitions to herbivory seem to have especially large effects on the microbiome. Mammals with independently evolved herbivorous diets host similar microbiota. Additionally, some of the same genes and pathways that differ in abundance between herbivorous and carnivorous microbiomes also rapidly shift in corresponding directions in humans who change from vegetarian to omnivorous diets [26]. Interestingly, insectivory has also been associated with some degree of convergence in mammalian microbiomes [46].

**Fig. 2.**
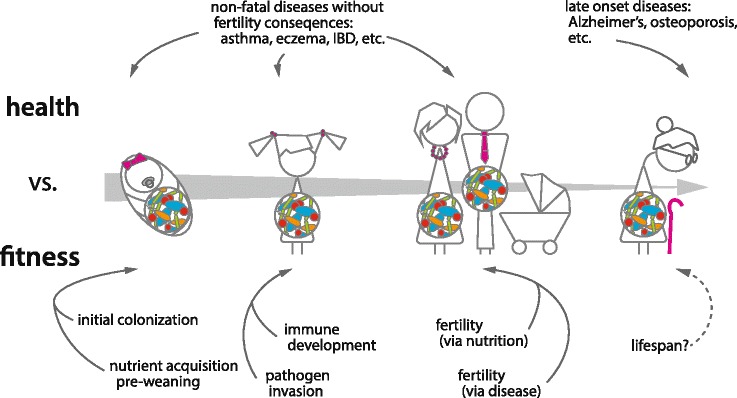
Host–microbiome interactions can affect both health and fitness. Dysbiosis is associated with a number of negative *health* outcomes, including obesity, asthma, and certain cancers. Negative health outcomes are not sufficient evidence for coevolution of the microbiome and host, however. Not all of these diseases result in negative *fitness* consequences by limiting reproductive success. Microbiomes potentially impact host *fitness* at multiple stages of life by affecting survival through reproductive years or reducing fertility. In infancy, microbes extract energy from non-digestible components of milk, increasing nutrient acquisition at this vulnerable age. During childhood, a stable microbiome prevents invasion of deadly pathogens. In adulthood, the microbiome potentially influences fertility, either by altering nutrition or causing disease. Finally, the microbiome may be important for lifespan. Although lifespan after menopause will not result in more children, the grandmother hypothesis predicts that care of extended kin results indirectly in higher fitness [139]. *IBD* inflammatory bowel disease

Major dietary transitions inevitably correlate with numerous other physiological changes, both related to and independent of diet itself, simply as a result of phylogenetic non-independence [47]. As a consequence, interpreting these changes as evidence of diet per se structuring the microbiome is likely an oversimplification. Numerous mammals host gut communities contrary to the general pattern of convergence by diet. Panda bears, despite being strict herbivores, host gut microbiomes comparatively similar to their carnivorous and omnivorous confamilials [35] and that differ substantially in functional gene complement and ecological dynamics from other herbivores [48, 49]. Work from our group shows that baleen whales, despite an entirely animal-based diet, host microbiomes that share similarities in both taxonomy and functional gene complement with the foregut-fermenting herbivores, to which they are distantly related [50]. Among bats, which have evolved numerous dietary specializations including nectivory, carnivory, sanguivory, frugivory, and insectivory, surveys revealed conflicting patterns with respect to diet and microbiomes (Fig. 2c) [51, 52]. Within species, dietary effects on the microbiome can be assessed independently of other factors. In comparative analyses, these effects must be understood in context with changes to things like gut morphology. Notably, both whales and pandas retain gut morphological similarities to their closest relatives that mirror the similarities in their microbiomes. Therefore, the relationship between diet, phylogeny, and the microbiome is not always a straightforward one.

## Consequences of altering species-specific microbiomes

**Fig. 3.**
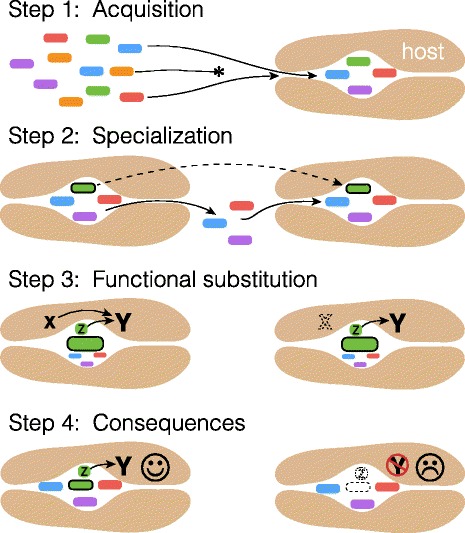
A non-adaptationist model for consequences of codiversification in microbiomes. In *Step 1*, a host lineage evolves permissive but variable filters for a gut microbiome, allowing diverse microbes to colonize its gut. In *Step 2*, a subset of microbes (*dark outline*) specialize in the host lineage, losing genes necessary to colonize diverse environments in favor of specialization on the particular host niche. As host genes creating this specific niche drift, the specialized microbes follow. In *Step 3*, the codiversifying microbes are now reliable environmental stimuli, and serve as developmental cues, reducing constraint on the host genome for essential processes. Mutations in the host genome arise that are neutral in the presence of these microbes, but deleterious in their absence. For example, an essential host-encoded developmental molecule *X* is required to signal *Y*. Microbial product *Z* elicits a similar downstream effect as *X*. At some point, a mutation in the host genome results in the loss of function of *X*, which is neutral when microbially encoded *Z* is present. In *Step 4*, in the absence of the codiversifying microbe, neither *X* nor *Z* is present to signal to *Y*, resulting in reduced fitness of the host

The mammalian gut microbiome clearly shows the deep imprint of mammalian evolutionary history, both at the level of community similarity and within individual microbial lineages. What are the consequences of these relationships? When does perturbation result in health consequences, and when are these consequences relevant to evolutionary fitness (Fig. 2)? Can the “microbiome” even be considered a thing that evolves (Box 3, Fig. 3)?

Growing evidence suggests shared evolutionary history matters to both hosts and microbiota. Although germ-free mice can be colonized by some bacteria from a wide range of environments, microbes from more closely related donor species often colonize more effectively [53]. Moreover, host-specificity per se affects the host: mice colonized with gut microbiota from humans, or even rats, fail to develop fully mature intestinal immunity, and are less protected from infection compared to mice with native microbiota [54]. Gut microbiota transplanted among multiple related species of mice show physiological effects that correlate with the evolutionary distance between the host and donor [55]. Host specificity affects colonization dynamics for some specific microbial lineages in the vertebrate gut, including *Lactobacillus* [56] and *Candidatus* Savagella, also known as segmented filamentous bacteria, which have potent immunomodulatory activity [57].

Correlations between shared evolutionary history and impact on mammalian hosts are also beginning to appear in broad comparative surveys across populations and species, although methods to formally examine cophylogenetic patterns in such datasets are still in their infancy. *H. pylori*, which has long been known to **codiversify** with human mitochondrial lineages [31], induces variable gastric disease symptom severity in different host genotypes in a manner consistent with either coevolutionary processes or lineage-specific adaptation [58]. In a recent paper seeking to tease apart signals of dietary and phylogenetic correlation in mammalian gut microbiota, many of the most tightly codiversifying microbial lineages also associated with inflammatory bowel disease in human datasets, suggesting that the pattern of codiversification itself may be an especially useful tool for finding microbes relevant to human health [59]. These codiversified lineages make clear candidates for investigating the underlying basis for recent formulations of the “hygiene hypothesis”, which posit that immune stimulation by microbes has a causal relationship with a number of chronic disease states, and that differences in transmission efficiency, coupled with the increased use of antibiotics, are a risk factor in some societies [23, 60].

## Mechanisms underlying species-specific microbiomes

As we have shown, perturbing species-specific microbiomes results in negative health and fitness consequences. Characterizing the mechanisms that determine these microbiome configurations is thus a key prerequisite to exploiting the microbiome therapeutically. Two broad categories of mechanisms underlie microbiome assembly: transmission and filtering.

Species specificity of individual microbial lineages arises via the restricted transmission of microbes within host species. Certain bacterial lineages in primates and across mammals more broadly show these patterns [59, 61]. Two types of transmission are possible, with different evolutionary implications: **vertical transmission**, where microbes pass directly from parents to offspring [62], and **horizontal transmission**, where microbes exchange non-filially. Strict vertical transmission leads to **cospeciation** of symbionts when host populations diverge, resulting in high fidelity of host-microbe relationships over long evolutionary time scales. This effect is notable in obligate intracellular microbes, which arose repeatedly in insects [63–65]. These intimate interactions can lead to **coevolution** between symbiont and host, leaving common signatures in symbiont genomes, including reduced genome size [66]. Although mammals lack intracellular microbe transmission, extracellular vertical transmission occurs through coprophagy and other mechanisms. In humans, mothers transfer microbes to offspring at birth and during nursing, as evidenced by strain sharing [67, 68].

Horizontal transmission decreases the evolutionary fidelity of host–microbe relationships. Vertical inheritance tends to take place early in life, when transferred microbes easily establish resident populations in offspring as primary colonizers. Microbes transferred later in life face more difficulty in permanently integrating into a stable complex community [69]. Horizontal transfer also occurs between species [39], homogenizing their microbiomes. Microbes spread horizontally in a number of different ways, including through coprophagy [70], social interactions [71–74], and cohabitation [75, 76].

Microbiomes adopt species-specific configurations through microbe filtering in the host. Lourens Baas Becking coined an enduring—albeit inaccurate—null hypothesis in microbial ecology: “Everything is everywhere, but the environment selects.” Even if this model is not universally true [77], it provides a useful frame for considering the myriad environmental microbes that contact host ecosystems, yet remain transient. Two processes lead to microbe filtering in host microbiomes: competition among microbes and habitat filtering [78, 79]. Phylogenetic patterns of co-occurrences and metabolic modeling of gut microbiomes indicate that habitat filtering plays a larger role in determining microbiome composition than does direct competition [80].

What factors underlie habitat filtering in the gut? Nutrient availability likely plays a key role, given the strong influence of host diet in shaping gut microbiomes [24, 26]. Many other physical and chemical factors differ in the gut between species, including types of digestive organs, digestive tract length, peristalsis rate, pH, oxygen levels, immune systems, and host derived molecules [81–88].

Genetic approaches help us understand which host factors influence microbiome composition, either through transmission or filtering. Host genetics determines the composition of the gut microbiome to some extent and the abundances of several common gut microbes are heritable in multiple human populations, including the family Christensenellaceae, genus *Methanobrevibacter*, and genus *Faecalibacterium*, as shown by several groups including our own [89–93]. Heritability in this case does not necessarily mean vertical inheritance from mother to offspring, although that occurs to some extent. Rather, inter-individual genetic variation correlates with the abundances of microbes in the gut, due to either transmission or filtering. Heritability studies in twins control for vertical transmission, as rates of vertical transmission are not assumed to differ between monozygotic twins compared to dizygotic twin pairs. For the microbes identified in these studies [89, 93], the heritability patterns presumably result from processes like filtering. In this case, filtering is probably driven on the host side by such biochemical and physical factors as gut pH, peristalsis rate, metabolite concentrations, and IgA levels, which vary in a heritable manner among individuals. Genome-wide association studies of the microbiome, although underpowered at current sample sizes, will pinpoint host pathways and processes that might play a role in filtering. To date, these studies implicate immune and diet/nutrient-related genes as important modulators of microbiome composition. [91, 93–96].

Different portions of the microbiome likely follow fundamentally different dynamics, complicating our ability to disentangle the competing effects of transmission and filtering across evolution [97]. Even when the similarity among gut communities precisely mirrors the evolutionary history of their hosts in a pattern of phylosymbiosis, as has been demonstrated for hominid apes [98], some members of the community may be determined via environmental filtering by phylogenetically correlated factors (like gut morphology), while others individually track the host phylogeny itself in a pattern of codiversification [32, 99]. Systematically incorporating phylogenetic information from gut microbes themselves into comparative datasets offers great promise for teasing apart the effects of different host factors on microbiomes [59]. If we can identify general patterns linking evolutionary processes to the roles played by members of the gut microbiome on host health, phylogenetic information may prove to be a useful tool for identifying and manipulating health-relevant microbes.

## Towards microbial medicine—open questions

Humans live in concert with the microbes around and inside our bodies, and have since our divergence from our most recent common ancestors. By interrogating the characteristics of and mechanisms underlying this divergence, we should be able to gain the most comprehensive view of what makes the human microbiome “human”, and which members of it are most relevant to different components of human health and fitness. Although the complexity of the interactions within the microbiome and between microbes and hosts presents a major challenge, a more concerted and predictive theoretical framework is imperative to progress [100]. Specifically, applying phylogenetic and population genetic approaches to query the targets and effects of natural selection in the microbiome will allow us to explicitly model the assembly and function of this inner microbial ecosystem in ways that allow us to move from the descriptive to the prescriptive. We are now poised to address several outstanding questions about the evolution of the human gut microbiome and use this information in medical research.

First, which taxa in the gut show high degrees of species specificity, and what mechanisms maintain this relationship? Shotgun **metagenomics** can provide strain-level insights into the microbiome [101]. Broadly sampling host species with convergent phenotypes, such as nocturnality, or environments, such as high altitude, would allow the assessment of filtering factors decoupled from phylogeny. Teasing apart these influences is relevant for medical microbiome research, because mode of acquisition likely affects our ability to use or target particular microbes therapeutically.

Second, can we develop a modeling framework for host–microbiome interactions to inform our baseline assumptions about microbial dynamics in the gut within and across generations (for example, see [102, 103])? Population genetics theory plays a pivotal role in defining expectations in evolutionary studies, including host–pathogen interactions [104–107]. Additionally, the community genetics framework assesses the effect of a particular organism’s heritable traits on the ecosystem more broadly [108]. There is strong motivation to model evolutionary processes of host–microbiome systems perhaps borrowing from some of these approaches.

Finally, to what degree are patterns of codiversification the result of adaptation, and to what degree can they be explained by neutral processes? It is often implied that humans and their microbes have adapted to each other, and that perturbing this relationship results in disease. Evidence suggesting non-neutral processes exists in a few cases [54, 58]. We must demonstrate fitness consequences in these cases when adaptation is assumed—and be open to non-adaptive explanations for health-relevant phenotypes (Box 3).

As we move forward, bringing these tools and this knowledge into medical microbiome research—taking an evolutionary medicine approach—can illuminate mechanisms underlying dysbiosis and allow us to harness the potential of the microbiome to improve human health.

## **Box 1** Definitions

- **Microbiome:** the biotic and abiotic habitat comprising the community of microorganisms associated with a particular environment or host. Sometimes synonymized with “microbiota”, though it has been argued the latter should be reserved for marker-gene-based descriptions of the biotic component of the microbiome [109]
- **Metagenome:** the collective genomes, or genomic components, of the microbiome. Empirically, metagenomes are investigated by random shotgun sequencing of the mixed community, rather than targeted sequencing of marker genes such as 16S rRNA.
- **Hologenome:** the genome of a host animal and its metagenome [110–112]. Conceived in light of a growing awareness of the importance of microorganisms to host organism biology, the “hologenome concept of evolution” considers the host and its microbes as an ecosystem, arguing that changes in phenotype due to exchange of symbiotic partners can be usefully analogized to changes in phenotype due to changes in genotype within an evolutionary framework [113, 114]. This concept is controversial [115], in part because imperfect vertical transmission of symbiotic components of the “hologenome” precludes analysis under standard models of evolution by natural selection.
- **Holobiont:** a host animal and its microbial associates. Two animals with identical genotypes but different microbial associates could express different “holobiont” phenotypes.
- **Co-evolution:** causally related, reciprocal changes in allele frequencies within populations of different organisms. This term has been used with conflicting or imprecise meanings for decades [116]; we prefer the usage outlined by Janzen [117], which is defined by a population-genetic *process*. Other previous uses have described *patterns*, which can be more usefully described with specific and discrete terms (e.g., “codiversification” or “phylosymbiosis”).
- **Cospeciation:** one particular *event* in evolutionary history, in which the speciation of one organism itself causes the contemporaneous speciation of another organism. Cospeciation events over time can produce a pattern of codiversification; horizontal transmission can make this harder to detect, and other processes of adaptation and transfer can also produce tightly codiversified phylogenies in the absence of cospeciation [118, 119].
- **Codiversification:** a cophylogenetic *pattern* in which biologically associated organisms display predicted evolutionary histories more similar to one another than expected by chance. We advocate the special use of the term “codiversification”, distinct from coevolution or cospeciation, as a precise way to refer to a particularly interesting and frequently observed pattern. Codiversification might arise from a history of cospeciation, but could also result, for example, from the shared experience of population vicariance across a landscape inhabited by both organisms.
- **Phylosymbiosis:** a phenetic *pattern* of congruence between the phylogeny of a clade of host organisms and the similarities of their symbiotic microbial communities [55]. Phylosymbiosis does not necessarily require the shared evolutionary history of microbiota and host, but can arise via structuring of microbial communities in ways correlated with host phylogeny [59, 99].
- **Vertical transmission:** acquisition of microbes directly from an organism’s parents. This conceivably happens either via transmission through the germline, as is frequently observed in insects, or subsequent interactions.
- **Horizontal transmission:** acquisition of microbes from sources other than an organism’s direct parents, such as the environment or from non-parental conspecifics.

## **Box 2** A glimpse into human microbiome evolution through ancient DNA

How can we know what our microbiomes looked like in the past? Were the microbiomes of our ancestors more diverse than our own, as predicted by the disappearing microbiome hypothesis [23]?

Although comparing non-Western to Western microbiomes offers insights into how modern diets and medicine shape microbiome composition, no living population today carries an “ancestral” microbiome. Rather, turning to ancient samples such as coprolites [120–122], dental calculus [123–125], tissue retrieved from permafrost [126, 127], and mummified remains [128, 129] offers us a glimpse into what our microbiomes looked like throughout the course of hominin evolution [130].

Although coprolites or mummified intestinal contents give us the best snapshots of ancient gut microbiomes, typically these samples preserve poorly and not all profiled samples contain what we think of as gut microbes [121].

Dental calculus, on the other hand, preserves quite well. Diversity of oral microbiomes decreased with the advent of agriculture, as observed from a series of samples dated between 7000 years ago to modern day [123]. Additionally, ancient oral microbiome samples carry both known opportunistic pathogens and, interestingly, putative antibiotic resistance genes [124].

Recently, sequencing of Neanderthal dental calculus samples from two caves revealed oral microbiomes that differed according to inferred meat eating behavior at each site. The microbiota of putatively meat-eating specimens closely resemble hunter-gatherer populations, while non-meat eating specimens resemble forager-gathering chimpanzees [125]. Given the explosion in ancient DNA research, it is likely we will know much more about pre-historic human microbiomes within the next several years.

## **Box 3** Do microbiomes evolve?

Microbiomes clearly impact host performance, and have changed along the branches of the animal evolutionary tree. Individual microbes, and their hosts, are clearly subject to evolutionary forces. But can a gut microbiome itself really be said to evolve?

Classically, evolution by natural selection requires three things: 1) phenotypic variation in a population, 2) differential reproductive success as a consequence of that variation, and 3) inheritance of said variation. Proponents of the **hologenome** concept of evolution argue that the parallels between phenotypes induced in an organism by genes in its genome and those induced by microbes in a combined host–microbe system, or **holobiont**, are strong – sufficiently so that host–microbe systems can usefully be examined as an extension of the same evolutionary frameworks built to explore descent and modification of single organisms [113, 114]. Critics have argued that differences in the modalities of inheritance of genes and microbes are too great for such analogies to be truly illuminating [61, 115]. Most would agree, however, that the variation in and fitness consequences of microbiome-related phenotypes highlights the importance of understanding the mechanisms underlying their effects across generations.

The most conceptually straightforward such mechanism—direct parental inheritance—may not be the most relevant to gut microbiomes. Extremely strict vertical transmission of microbes from parent to offspring would be necessary to link host and microbial genotypes sufficiently tightly for selection on one generation to change “holotype” frequency in the next. Some gut microbiomes may meet this criterion—in turtle ants, for example, gut bacteria are passed on to new adults, which then immediately grow a literal filter in their gut capable of keeping other microbes out [131]; some true bugs (Hemiptera: Alydidae) use a specialized organ to deposit gut symbionts atop their eggs, ensuring they are the first things consumed by the emerging young [132]. The exquisite level of behavioral and morphological specialization evidenced in these systems suggests the difficulty of maintaining partnerships over time. For most systems, heritability is unlikely to be strong enough for the microbiome itself, in any meaningful sense, to evolve.

In a recent perspective, Doolittle and Booth [133] argue that particular metabolic roles, rather than the potentially diverse casts of microbes performing them, are the units most relevant to natural selection in the microbiome. This framing extends an informal model that may provide a more intuitive point of entry for discussing evolution in complex or dynamic microbiomes [113]. By focusing on the microbial products most likely to be directly perceived by a host, it tries to account for the observation that, while specific microbes are highly variable among microbiomes, the representation of putative functional pathways appears to be much more consistent, suggesting some level of redundancy. This model may be particularly appropriate for situations where the relevant metabolic pathways are directly related to putative microbiome function, such as processing of complex polysaccharides in plant-based diets.

Focusing on roles rather than players may also help in constructing alternative models to understand the fitness consequences of microbial associates that do show some degree of conservation through time—including the species-specific (or even codiversifying) microbes described from some mammalian guts. While the above model primarily interprets evolution in the microbiome in the context of host- (or holobiont-) level adaptation, real fitness consequences could also arise purely via random fixation of nearly neutral mutations in hosts and sets of species-specific microbes (Fig. 3). Rather than performing some particular metabolic function, the “role” performed by these microbes might be something like serving as a developmental cue—but would still result in fitness defects if absent.
